# Supplementary material for: Bioavailable phosphite in the surface ocean during the Great Oxidation Event
Source: Nat Commun. 2025 May 24;16:4825. doi: 10.1038/s41467-025-59963-0 (PMC12102248; doi:10.1038/s41467-025-59963-0)
Supplement: Supplementary file 1 — Supplementary Information [file 41467_2025_59963_MOESM1_ESM.pdf]

Supplementary Material to

Bioavailable phosphite in the surface ocean during the Great Oxygenation Event

Abu Saeed Baidya*[1], Joanne S. Boden[1], Yuhao Li[2], Albertus J. B. Smith[3,4], Kurt Konhauser[2,4], Eva E. Stüeken[1],

[1]School of Earth and Environmental Sciences, University of St. Andrews, St Andrews KY16 9TS, United Kingdom

[2]Department of Earth and Atmospheric Sciences, University of Alberta, Earth Sciences Building, Edmonton, Alberta T6G 2E3, Canada

[3]DSTI-NRF Cimera and PPM Research Group, Department of Geology, University of Johannesburg, Auckland Park, 2092, Johannesburg, South Africa

[4]Dept of Earth & Environmental Sciences, University of Manchester, Manchester M13 9PL, United Kingdom

**Corresponding author; email id.: asb27@st-andrews.ac.uk*

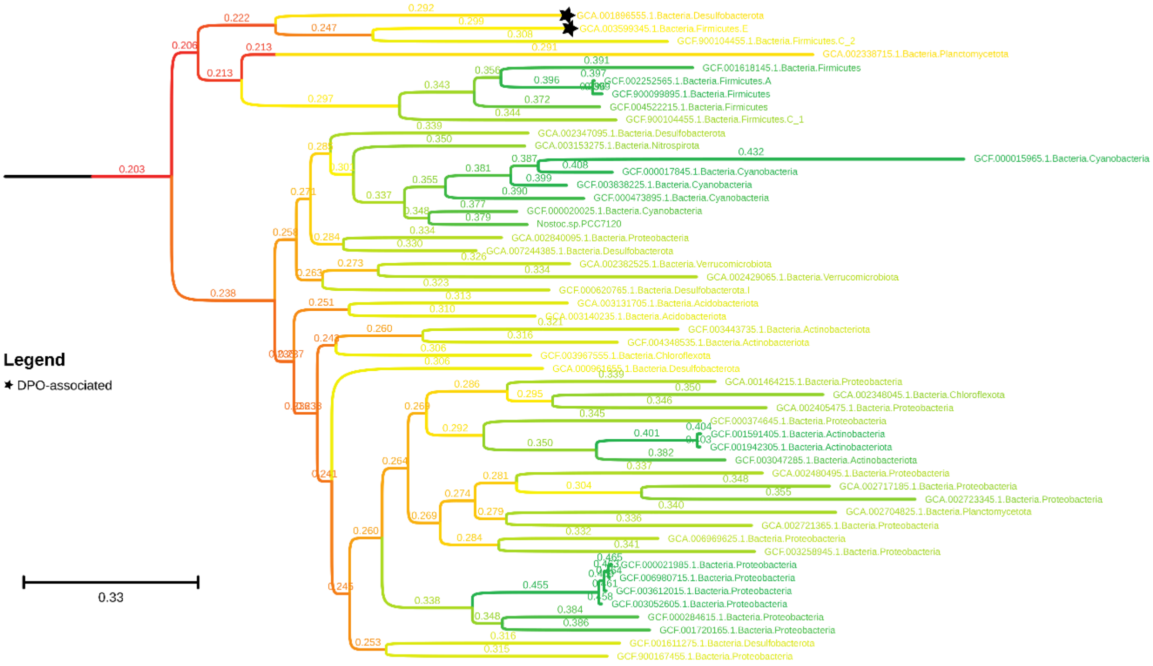

Figure S1: Evolutionary tree of the enzyme responsible for microbial phosphite oxidation (namely phosphite dehydrogenase; PtxD). *Enzymes found in organisms with genes for dissimilatory phosphite oxidation (DPO) are annotated with black stars, whereas all others are associated with assimilatory phosphite oxidation due to the lack of genes known to be required for DPO (namely one or more of ptxE, ptdC, ptdG, ptdH, ptdI and ptdF). Numbers on branches and their colours represent ancestor deviation values estimated by MAD[60] . They indicate where the tree is rooted, so values closer to zero (in red colours) are more likely to represent the tree's root. Leaf labels describe the microbial organism within which the phosphite oxidation gene was found in the format; GTDB release 95 identification number '.' Domain '.' Phylum. The scale bar represents the average number of amino acid substitutions per site.*

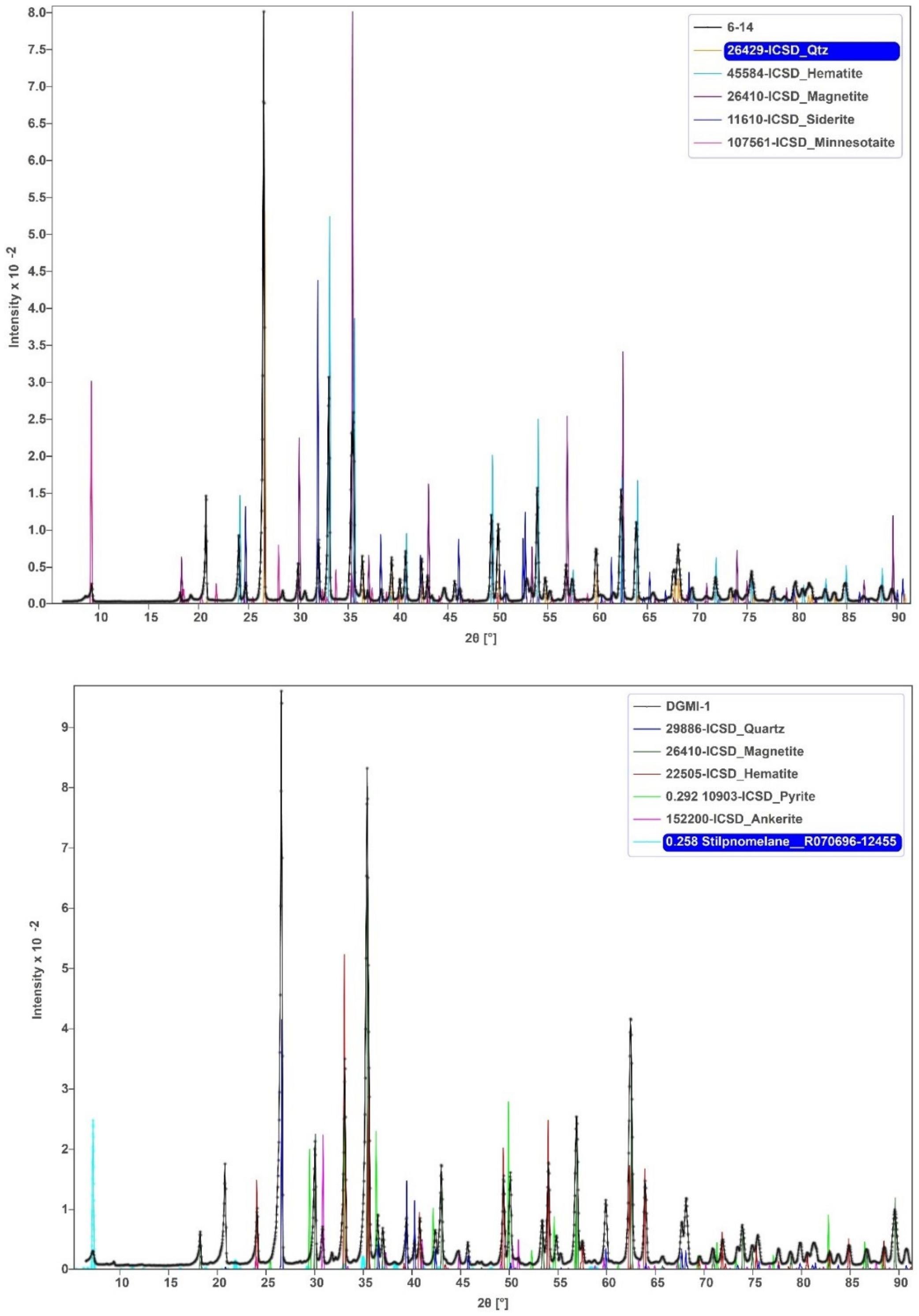

Figure S2: Typical XRD patterns of Kuruman (top) and Dales Gorge (bottom) samples. *The XRD pattern of the banded iron formation samples is shown by black line. In the bottom Heights of the pyrite and stilponomelane peaks are diminished for better visualization. The patterns of the minerals are taken from Inorganic Crystal Structure Database (ICSD) database. The numbers shown in the legend are the ICSD code of the respective minerals. The pattern matching is done by the CrystalDiffract software.*

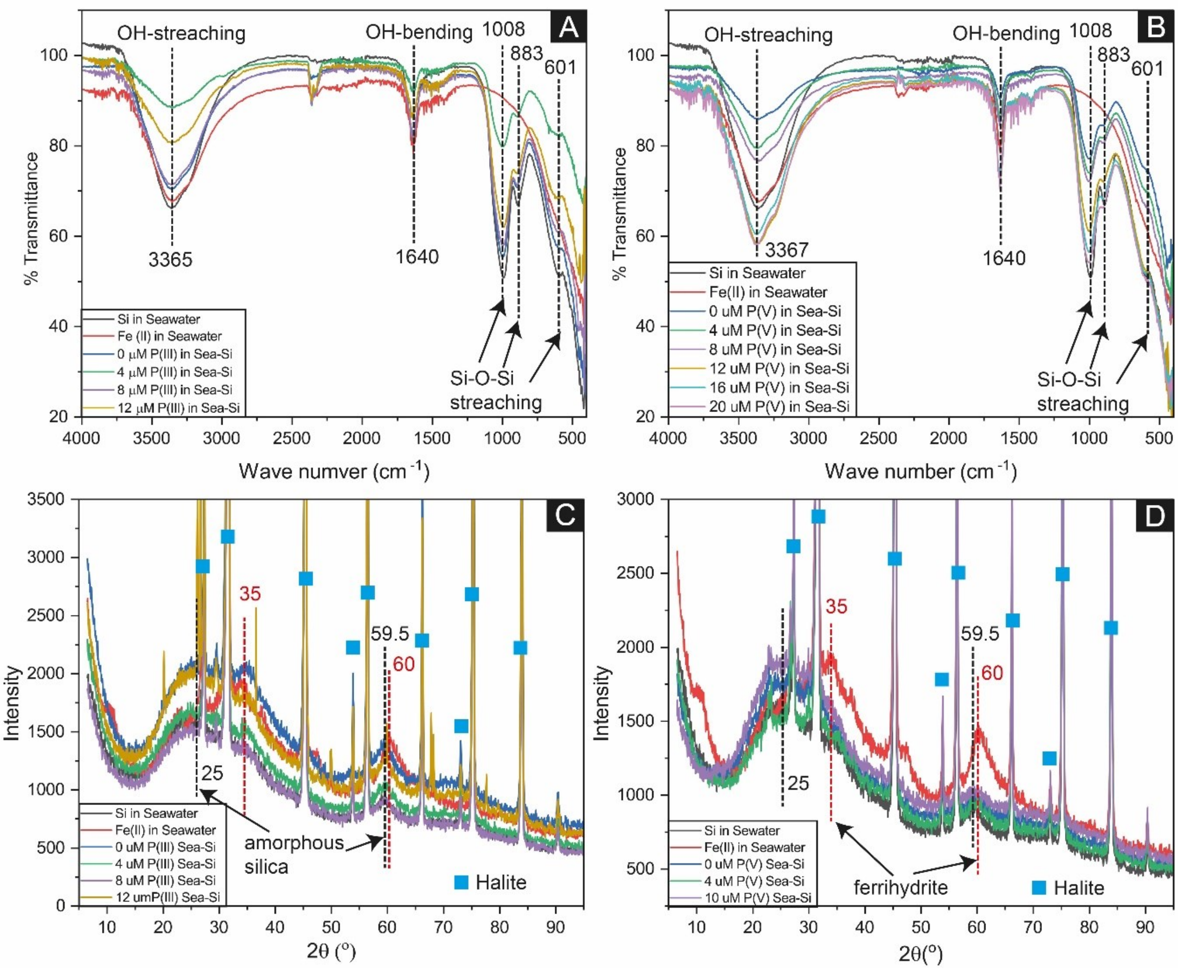

Figure S3: Representative Fourier transform infrared spectroscopy (A, B) and X-ray diffraction (C, D) data of precipitates from different experiments. *In 'Si in Seawater' and' Fe(II) in Seawater' experiments, Si and Fe (II) were added to the seawater solutions. The precipitates had white and red/reddish brown color, respectively. Adding only Si and only Fe(II) in seawater precipitate silica and HFO (most likely ferrihydrite), respectively (C, D). Broad peaks of precipitated solids in these two experiments suggest that they are amorphous (C, D). Both these phases have OH in their structures as seen in FTIR data in A and B, suggesting that both phases are hydrous. 'Sea-Si' stands for seawater with 2.2 mM Si. FTIR pattern of the adsorption experimental samples is dominated by amorphous silica. In C and D, halite (NaCl, blue squares) is identified, however, it has precipitated during freeze-drying of the experimental precipitates. The concentration of NaCl (0.56M) is not enough to precipitate halite during the experiment. In P(III) adsorption experiments, both amorphous silica and HFO are identified (C).*

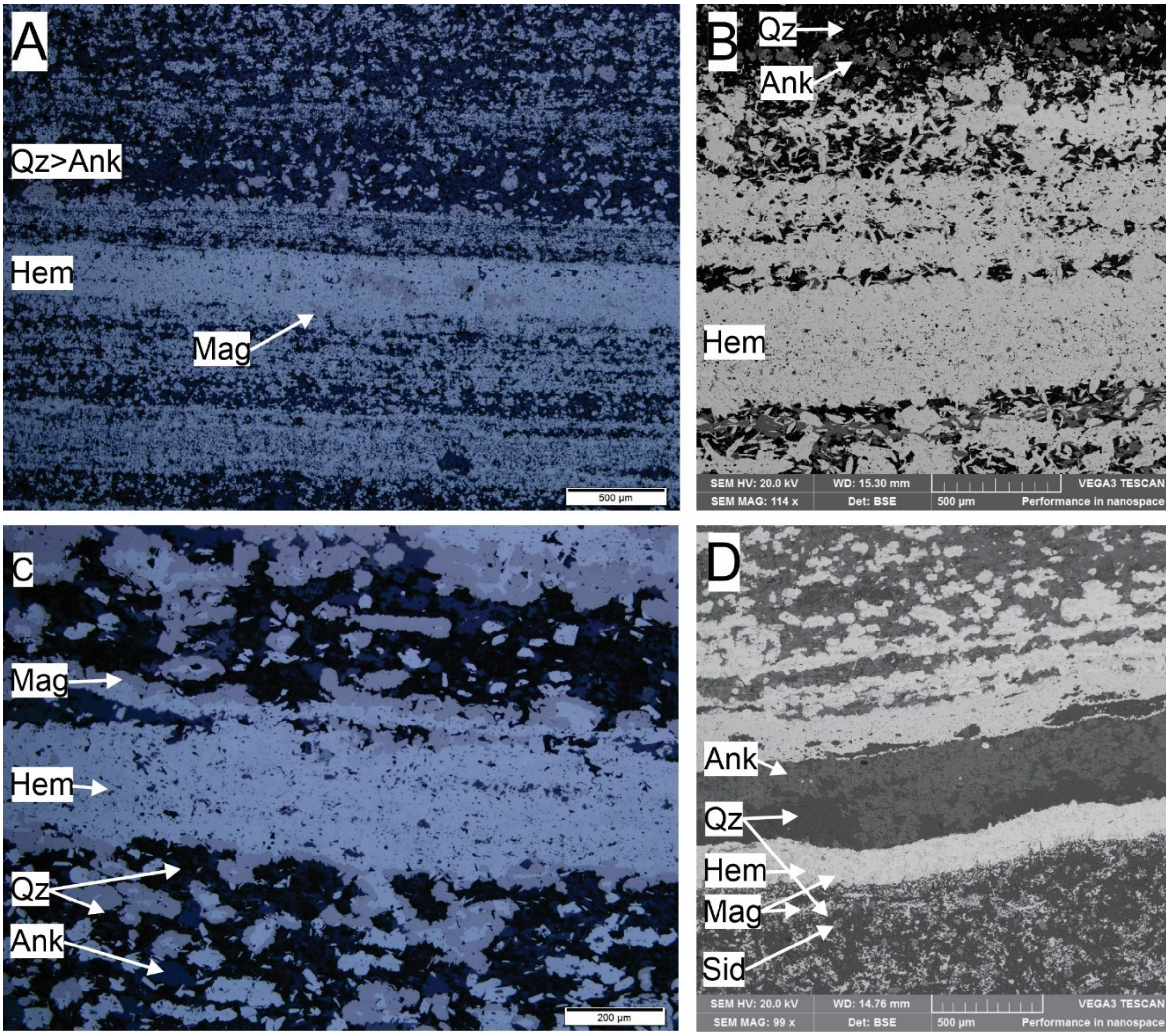

Figure S4: Reflected light optical microscopy photomicrographs (A and C) and scanning electron microscope backscatter electron (SEM-BSE) (B and D) illustrating the mineralogy some of the studied geological samples from the Kuruman Iron Formation. *A) BIF depicting microbands of very fine hematite (Hem) with minor, coarser magnetite (Mag) alternating with microbands of quartz with minor ankerite (Ank), magnetite and hematite (sample 6/100; Whitebank Member oxide-carbonate facies BIF). B) Microband composed of near monomineralic, fine-grained hematite occurring directly below a quartz and ankerite microband (sample 6/100). C) Microband of near monomineralic, fine-grained hematite rimmed by coarser magnetite alternating with microbands of mixed quartz-ankerite-magnetite with hematite inclusions in the magnetite (sample 6/14; Derby Member oxide-carbonate facies BIF). D) Microbands of near monomineralic, fine-grained hematite with lesser magnetite alternating with microbands of quartz-ankerite with minor magnetite and siderite (Sid) (sample 6/14).*

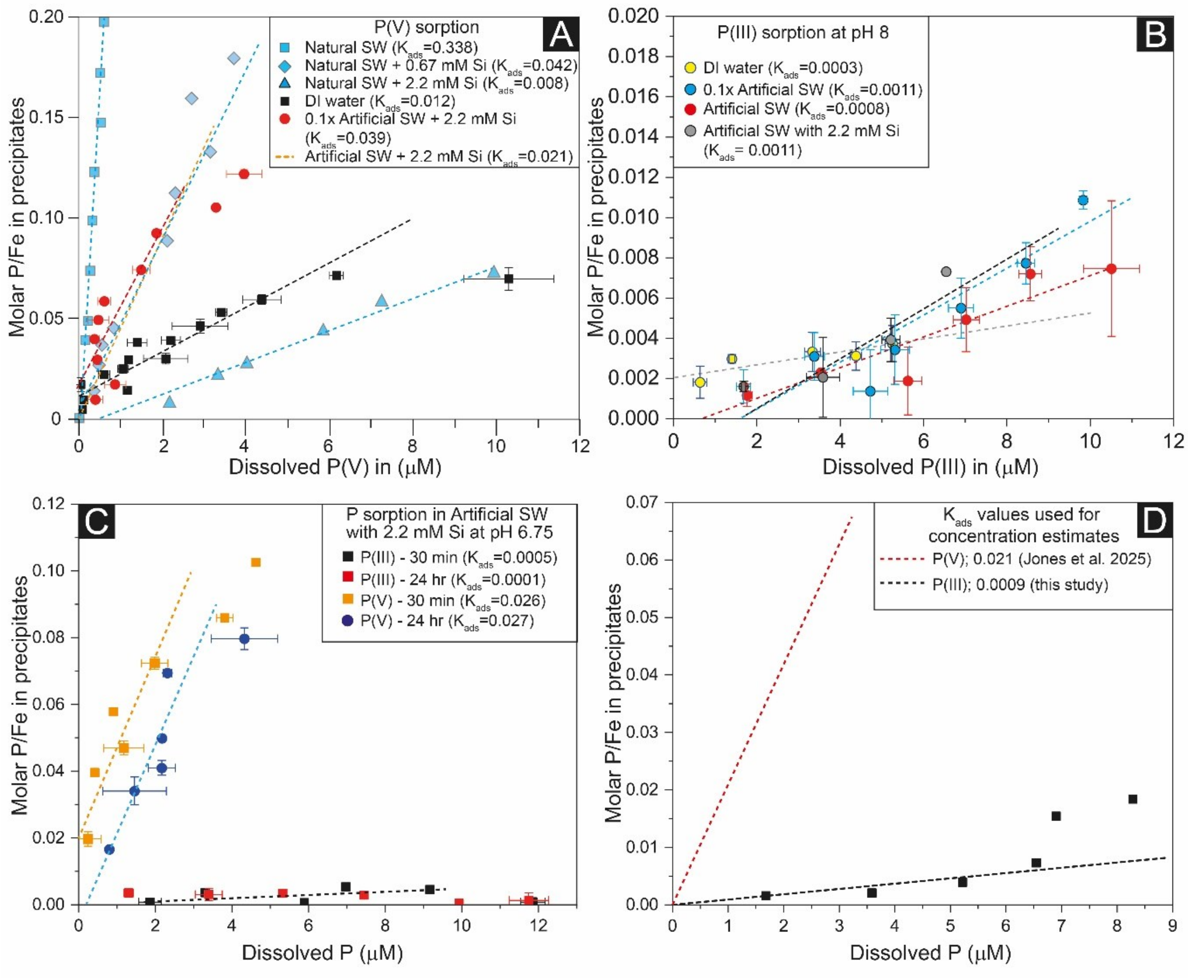

Figure S5: Complete dataset for Seawater with Si experiments and the data is compared to other datasets. *(A) Compiled P(V) sorption data from previous studies (ref. 28) are compared with new data (black and red). (B-C) P(III) adsorption data generated in this study. (D)* K_{ads} *values used for P(V) and P(III) estimates in seawater around the Neoarchean-Paleoproterozoic boundary. We have not considered last two datapoints with P/Fe values > 0.01 in D because we suspected contamination during our experiments. For additional details, see Fig. 1 in the main text.*
