# Supplementary material for: Bioavailable phosphite in the surface ocean during the Great Oxidation Event
Source: Nat Commun. 2025 May 24;16:4825. doi: 10.1038/s41467-025-59963-0 (PMC12102248; doi:10.1038/s41467-025-59963-0)
Supplement: Supplementary file 2 — Description of Additional Supplementary Files [file 41467_2025_59963_MOESM2_ESM.pdf]

Description of Additional Supplementary Files:

Supplementary Dataset 1: Adsorption test data

Supplementary Dataset 2: P speciation in BIF

Supplementary Dataset 3: FTIR data

Supplementary Dataset 4: XRD data
